# Supplementary figures and images for: Assessing the impact of preventive mass vaccination campaigns on yellow fever outbreaks in Africa: A population-level self-controlled case series study
Source: PLoS Med. 2021 Feb 18;18(2):e1003523. doi: 10.1371/journal.pmed.1003523 (PMC7932543; doi:10.1371/journal.pmed.1003523)

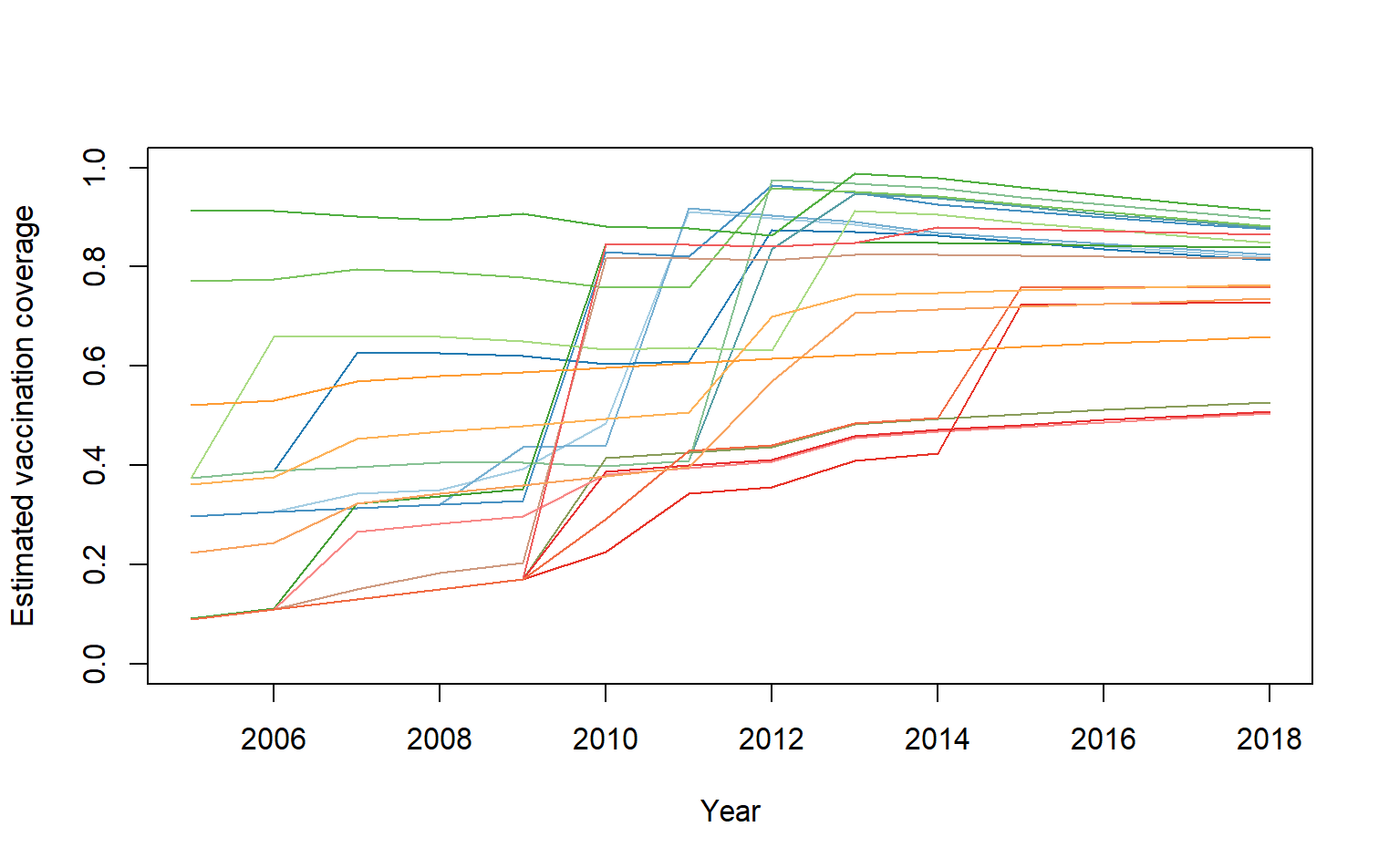

Supplement: S1 Fig — Each province is represented by a unique color. (TIFF) [file pmed.1003523.s001.tiff]

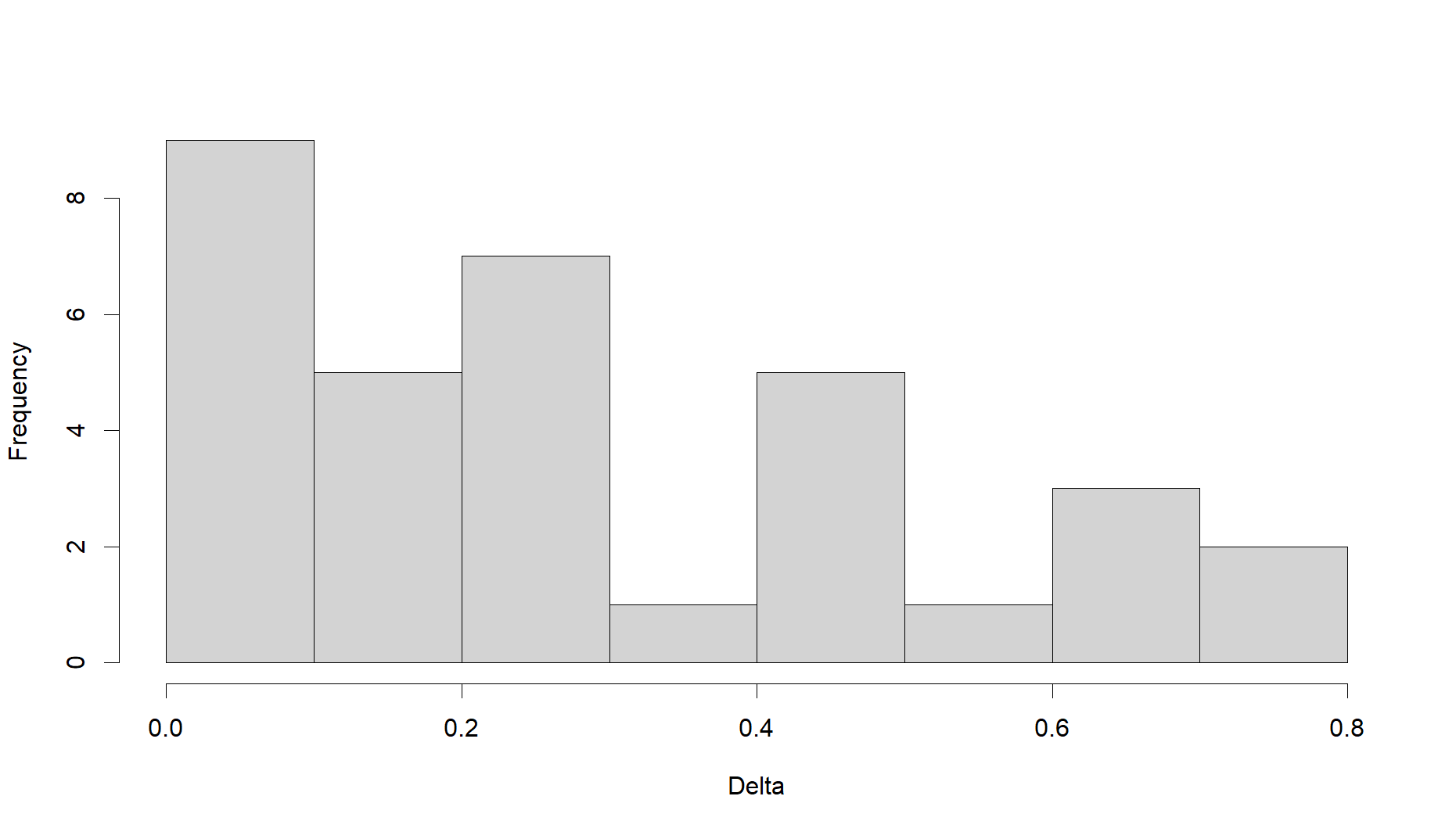

Supplement: S2 Fig — PMVC, preventive mass vaccination campaign. (TIFF) [file pmed.1003523.s002.tiff]
