# Supplementary material for: Assessing the impact of preventive mass vaccination campaigns on yellow fever outbreaks in Africa: A population-level self-controlled case series study
Source: PLoS Med. 2021 Feb 18;18(2):e1003523. doi: 10.1371/journal.pmed.1003523 (PMC7932543; doi:10.1371/journal.pmed.1003523)
Supplement: S1 STROBE Checklist — (DOCX) [file pmed.1003523.s003.docx]

STROBE Statement—checklist of items that should be included in reports of observational studies

|  | Item No | Recommendation |
| --- | --- | --- |
| **Title and abstract** | 1 | (*a*) Indicate the study’s design with a commonly used term in the title or the abstract Yes, see title |
|  |  | (*b*) Provide in the abstract an informative and balanced summary of what was done and what was found Yes, see abstraact |
| Introduction | | |
| Background/rationale | 2 | Explain the scientific background and rationale for the investigation being reported  Introduction section, paragraphs 1 to 4 |
| Objectives | 3 | State specific objectives, including any prespecified hypotheses Introduction section paragraphs 5, and Methods section, “Study hypothesis”, sub-section |
| Methods | | |
| Study design | 4 | Present key elements of study design early in the paper Introduction section paragraphs 5 |
| Setting | 5 | Describe the setting, locations, and relevant dates, including periods of recruitment, exposure, follow-up, and data collection Methods section, “Data used” sub-section |
| Participants | 6 | (*a*) *Cohort study*—Give the eligibility criteria, and the sources and methods of selection of participants. Describe methods of follow-up Methods section, “Analysis using the cohort design” sub-section  *Case-control study*—Give the eligibility criteria, and the sources and methods of case ascertainment and control selection. Give the rationale for the choice of cases and controls Methods section, “Mains SCCS analysis” sub-section  *Cross-sectional study*—Give the eligibility criteria, and the sources and methods of selection of participants |
|  |  | (*b*) *Cohort study*—For matched studies, give matching criteria and number of exposed and unexposed  *Case-control study*—For matched studies, give matching criteria and the number of controls per case |
| Variables | 7 | Clearly define all outcomes, exposures, predictors, potential confounders, and effect modifiers. Give diagnostic criteria, if applicable Methods section, “Mains SCCS analysis” sub-section |
| Data sources/ measurement | 8* | For each variable of interest, give sources of data and details of methods of assessment (measurement). Describe comparability of assessment methods if there is more than one group Methods section, “Data used” sub-section |
| Bias | 9 | Describe any efforts to address potential sources of bias Introduction section, paragraph 4, Methods, “Alternative SCCS models and sensitivity analyses” sub-section |
| Study size | 10 | Explain how the study size was arrived at Methods section, “Data used” sub-section |
| Quantitative variables | 11 | Explain how quantitative variables were handled in the analyses. If applicable, describe which groupings were chosen and why Methods, “Alternative SCCS models and sensitivity analyses” sub-section |
| Statistical methods | 12 | (*a*) Describe all statistical methods, including those used to control for confounding Methods, sub-sections “Mains SCCS analysis” , “Alternative SCCS models and sensitivity analyses” and “Analysis using the cohort design” |
|  |  | (*b*) Describe any methods used to examine subgroups and interactions Methods, “Alternative SCCS models and sensitivity analyses” sub-section |
|  |  | (*c*) Explain how missing data were addressed Methods, “Alternative SCCS models and sensitivity analyses” sub-section (paragraph 3) |
|  |  | (*d*) *Cohort study*—If applicable, explain how loss to follow-up was addressed  *Case-control study*—If applicable, explain how matching of cases and controls was addressed  *Cross-sectional study*—If applicable, describe analytical methods taking account of sampling strategy Not applicable |
|  |  | (*e*) Describe any sensitivity analyses Methods, “Alternative SCCS models and sensitivity analyses” sub-section |

Continued on next page

| Results | | |
| --- | --- | --- |
| Participants | 13* | (a) Report numbers of individuals at each stage of study—eg numbers potentially eligible, examined for eligibility, confirmed eligible, included in the study, completing follow-up, and analysed Results section, “Outbreak occurrence and PMVCs” sub-section |
|  |  | (b) Give reasons for non-participation at each stage Results section, “Outbreak occurrence and PMVCs” sub-section, paragraph 1 |
|  |  | (c) Consider use of a flow diagram Not relevant here |
| Descriptive data | 14* | (a) Give characteristics of study participants (eg demographic, clinical, social) and information on exposures and potential confounders Results section, “Outbreak occurrence and PMVCs” sub-section |
|  |  | (b) Indicate number of participants with missing data for each variable of interest Not relevant here |
|  |  | (c) *Cohort study*—Summarise follow-up time (eg, average and total amount) |
| Outcome data | 15* | *Cohort study*—Report numbers of outcome events or summary measures over time |
|  |  | *Case-control study—*Report numbers in each exposure category, or summary measures of exposure Results section, “Outbreak occurrence and PMVCs” sub-section, Table 2 |
|  |  | *Cross-sectional study—*Report numbers of outcome events or summary measures |
| Main results | 16 | (*a*) Give unadjusted estimates and, if applicable, confounder-adjusted estimates and their precision (eg, 95% confidence interval). Make clear which confounders were adjusted for and why they were included Table 2, Supplementray Tables S6 and S7 |
|  |  | (*b*) Report category boundaries when continuous variables were categorized Table 2 |
|  |  | (*c*) If relevant, consider translating estimates of relative risk into absolute risk for a meaningful time period Not relevant here |
| Other analyses | 17 | Report other analyses done—eg analyses of subgroups and interactions, and sensitivity analyses Results section, “Sensitivity analysis” sub-section |
| Discussion | | |
| Key results | 18 | Summarise key results with reference to study objectives Discussion section, paragraph 1 |
| Limitations | 19 | Discuss limitations of the study, taking into account sources of potential bias or imprecision. Discuss both direction and magnitude of any potential bias Discussion section, paragraph 7 |
| Interpretation | 20 | Give a cautious overall interpretation of results considering objectives, limitations, multiplicity of analyses, results from similar studies, and other relevant evidence Discussion section paragraphs 2 to 6 |
| Generalisability | 21 | Discuss the generalisability (external validity) of the study results Discussion section paragraph 8 |
| Other information | | |
| Funding | 22 | Give the source of funding and the role of the funders for the present study and, if applicable, for the original study on which the present article is based provided in the submission material |

*Give information separately for cases and controls in case-control studies and, if applicable, for exposed and unexposed groups in cohort and cross-sectional studies.

**Note:** An Explanation and Elaboration article discusses each checklist item and gives methodological background and published examples of transparent reporting. The STROBE checklist is best used in conjunction with this article (freely available on the Web sites of PLoS Medicine at http://www.plosmedicine.org/, Annals of Internal Medicine at http://www.annals.org/, and Epidemiology at http://www.epidem.com/). Information on the STROBE Initiative is available at www.strobe-statement.org.
