# Supplementary material for: Assessing the impact of preventive mass vaccination campaigns on yellow fever outbreaks in Africa: A population-level self-controlled case series study
Source: PLoS Med. 2021 Feb 18;18(2):e1003523. doi: 10.1371/journal.pmed.1003523 (PMC7932543; doi:10.1371/journal.pmed.1003523)
Supplement: S1 Table — The simulated counts were obtained from 10,000 random realizations of a Poisson process of rate λ = 96/479, based on the total number of outbreaks observed among the sample of 479 provinces over the study period. (DOCX) [file pmed.1003523.s004.docx]

**S1 Table:** Fit of the Poisson probability distribution to outbreak data.

| **Number of outbreaks** | **0** | **1** | **2** | **3** |
| --- | --- | --- | --- | --- |
| **Observed count** | 398 | 69 | 9 | 3 |
| **Simulated counts** : Median and 95% Confidence interval | 392 (375-408) | 78 (63-95) | 8 (3-14) | 0 (0-2) |

**S1 Table:** Fit of the Poisson probability distribution to outbreak data. The simulated counts were obtained from 10,000 random realizations of a Poisson process of rate λ = 96/479, based on the total number of outbreaks observed among the sample of 479 provinces over the study period.
