# Supplementary material for: Assessing the impact of preventive mass vaccination campaigns on yellow fever outbreaks in Africa: A population-level self-controlled case series study
Source: PLoS Med. 2021 Feb 18;18(2):e1003523. doi: 10.1371/journal.pmed.1003523 (PMC7932543; doi:10.1371/journal.pmed.1003523)
Supplement: S2 Table — (DOCX) [file pmed.1003523.s005.docx]

**S2 Table.** Correspondence table of provinces ISO codes from Fig 2 in the main text and complete country and province names.

| **Province ISO code** | **Country** | **Province** |
| --- | --- | --- |
| CAF_12 | Central African Republic | Ombella-M'Poko |
| CAF_14 | Central African Republic | Ouham-Pendé |
| CAF_5 | Central African Republic | Haute-Kotto |
| CIV_14 | Côte d'Ivoire | Savanes |
| CIV_17 | Côte d'Ivoire | Vallée du Bandama |
| CIV_18 | Côte d'Ivoire | Worodougou |
| CIV_19 | Côte d'Ivoire | Zanzan |
| CIV_6 | Côte d'Ivoire | Fromager |
| CIV_9 | Côte d'Ivoire | Lagunes |
| CMR_1 | Cameroon | Adamaoua |
| CMR_10 | Cameroon | Sud |
| CMR_2 | Cameroon | Centre |
| CMR_3 | Cameroon | Est |
| CMR_5 | Cameroon | Littoral |
| CMR_7 | Cameroon | Nord |
| CMR_8 | Cameroon | Ouest |
| CMR_9 | Cameroon | Sud-Ouest |
| GHA_1 | Ghana | Ashanti |
| GHA_2 | Ghana | Brong Ahafo |
| GHA_7 | Ghana | Upper East |
| GHA_8 | Ghana | Upper West |
| GIN_3 | Guinea | Faranah |
| GIN_4 | Guinea | Kankan |
| GIN_8 | Guinea | Nzérékoré |
| LBR_12 | Liberia | Nimba |
| MLI_5 | Mali | Koulikoro |
| MLI_8 | Mali | Sikasso |
| NGA_26 | Nigeria | Nassarawa |
| SDN_2 | Sudan | Darfur |
| SDN_5 | Sudan | Kordofan |
| SEN_10 | Senegal | Thiès |
| SEN_9 | Senegal | Tambacounda |
| TGO_3 | Togo | Maritime |

**S2 Table.** Correspondence table of provinces ISO codes from Fig 2 in the main text and complete country and province names.
