# Supplementary material for: Assessing the impact of preventive mass vaccination campaigns on yellow fever outbreaks in Africa: A population-level self-controlled case series study
Source: PLoS Med. 2021 Feb 18;18(2):e1003523. doi: 10.1371/journal.pmed.1003523 (PMC7932543; doi:10.1371/journal.pmed.1003523)
Supplement: S3 Table — IRR, incidence rate ratio; PMVC, preventive mass vaccination campaign. (DOCX) [file pmed.1003523.s006.docx]

**S3 Table.** Sensitivity of the self-controlled case-series method results to the imputation of the missing dates of events (outbreak) or exposure (Preventive mass vaccination campaign, PMVC).

| **Model** | **Imputed date of outbreak when missing (within the same year)** | **Imputed date of PMVC when missing (within the same year)** | **Exposure category** | **Number of events** | **IRR** | **95% confidence interval** |
| --- | --- | --- | --- | --- | --- | --- |
| SCCS Model 1 (main analysis) | July, 1st | Dec, 31st | Unexposed (Ref.)  Exposed | 26  7 | 1.00  0.14 | -  0.06-0.34 |
| Sensitivity analysis #1 | July, 1st | Jan, 1st | Unexposed (Ref.)  Exposed | 23  10 | 1.00  0.16 | -  0.07-0.36 |
| Sensitivity analysis #2 | Dec, 31st | Jan, 1st | Unexposed (Ref.)  Exposed | 21  12 | 1.00  0.22 | -  0.10-0.48 |
| Sensitivity analysis #3 | Jan, 1st | Dec, 31st | Unexposed (Ref.)  Exposed | 26  7 | 1.00  0.14 | -  0.06-0.34 |
|  |  |  |  |  |  |  |

**S3 Table.** Sensitivity of the self-controlled case-series method results to the imputation of the missing dates of events (outbreak) or exposure (Preventive mass vaccination campaign, PMVC). IRR: incidence rate ratio.
