# Supplementary material for: Assessing the impact of preventive mass vaccination campaigns on yellow fever outbreaks in Africa: A population-level self-controlled case series study
Source: PLoS Med. 2021 Feb 18;18(2):e1003523. doi: 10.1371/journal.pmed.1003523 (PMC7932543; doi:10.1371/journal.pmed.1003523)
Supplement: S4 Table — IRR, incidence rate ratio. (DOCX) [file pmed.1003523.s007.docx]

**S4 Table.** Sensitivity of the self-controlled case-series method results to the choice of start and end dates of the study period. IRR: incidence rate ratio.

| **Model** | **Beginning of the observation period** | **End of the observation period** | **Exposure category** | **Number of events** | **IRR*** | **95% confidence interval** | **Number of outbreak prevented** | **Total number of outbreak observed on the period** | **Percentage of outbreaks prevented** |
| --- | --- | --- | --- | --- | --- | --- | --- | --- | --- |
| SCCS Model 1 (main analysis) | Jan, 1^st^ 2005 | Dec, 31^st^ 2018 | Unexposed (Ref.)  Exposed | 26  7 | 1.00  0.14 | -  0.06-0.34 | 50 (28 to 80) | 96 | 34% (22% to 45%) |
| Sensitivity analysis #1 | Jan, 1^st^ 2007 | Dec, 31^st^ 2018 | Unexposed (Ref.)  Exposed | 17  7 | 1.00  0.28 | -  0.11-0.70 | 30 (10 to 59) | 79 | 28% (12% to 43%) |
| Sensitivity analysis #2 | Jan, 1^st^ 2005 | Dec, 31^st^ 2014 | Unexposed (Ref.)  Exposed | 26  6 | 1.00  0.12 | -  0.05-0.29 | 24 (10 to 42) | 61 | 29% (14% to 41%) |
|  |  |  |  |  |  |  |  |  |  |

**S4 Table.** Sensitivity of the self-controlled case-series method results to the choice of start and end dates of the study period. IRR: incidence rate ratio.
