# Supplementary material for: Assessing the impact of preventive mass vaccination campaigns on yellow fever outbreaks in Africa: A population-level self-controlled case series study
Source: PLoS Med. 2021 Feb 18;18(2):e1003523. doi: 10.1371/journal.pmed.1003523 (PMC7932543; doi:10.1371/journal.pmed.1003523)
Supplement: S1 Text — (DOCX) [file pmed.1003523.s008.docx]

**Assessing the impact of preventive mass vaccination campaigns on yellow fever outbreaks in Africa : a population-level self-controlled case-series study**

*Kévin Jean^1,2,3*^, Hanaya Raad^1,2,4^, Katy A. M. Gaythorpe^3^, Arran Hamlet^3^, Judith E. Mueller^2,4^, Dan Hogan^5^, Tewodaj Mengistu^5^*, *Heather J. Whitaker^6,7^, Tini Garske^3^, Mounia N. Hocine^1,2^*

*^1^ Laboratoire MESuRS, Conservatoire national des Arts et Métiers, Paris, France*

*^2^ Unité PACRI, Institut Pasteur, Conservatoire National des Arts et Métiers, Paris, France*

*^3^ MRC Centre for Global Infectious Disease Analysis, Department of Infectious Disease Epidemiology, Imperial College London, United Kingdom*

*^4^ EHESP French School of Public Health, Paris, France*

*5 Gavi, the Vaccine Alliance, Geneva, Switzerland*

*^6^ Statistics, Modelling and Economics Department, National Infection Service, Public Health England, Colindale, London, United Kingdom*

*^7^ Department of Mathematics & Statistics, The Open University, Milton Keynes, United Kingdom*

***Corresponding author:** [kevin.jean@lecnam.net](mailto:kevin.jean@lecnam.net)

**S1 Text : Cohort models and adjustment**

In a cohort design, the choice of covariates to include is critical to prevent bias due to residual confounding. As no clear consensus has emerged on the demographical and environmental drivers of yellow fever, we considered two (partially overlapping) sets of covariates that were previously used to reproduce the occurrence of yellow fever records in Africa at the province level. The first model is a statistical model reproducing the spatial distribution of yellow fever records with no explicit aims at reproducing underlying biological processes. The second model is a mechanistic model that aimed at reproducing the spatial distribution of the disease while including these processes, here the temperature-dependence of the yellow fever virus cycle. Variables included in each model are presented in Table A.

| Variable | Data source | Statistical model [1] | Mechanistic model [2] |
| --- | --- | --- | --- |
| **Human population size** (log-transformed) | [3,4] | X | X |
| **Proxy for surveillance quality**: country-level per capita rate of reporting suspected cases of fever and jaundice | Yellow Fever Surveillance Database, surveillance database established by the African Regional Office of WHO | X | X |
| **Longitude** | [5] | X |  |
| **Land cover type** | [6] | X |  |
| **Enhanced Vegetation Index:** optimised remote-sensing measure of vegetation | [7] | X | X |
| **Rainfall** | [8] |  | X |
| **Temperature suitability index** | [2] |  | X |

**Table A:** Covariates entered for the statistical and mechanistic models used in the cohort-style analysis measuring the association between the implementation of preventive mass vaccination campaign and yellow fever outbreak.

***Exposure models for candidate confounding factors***

Univariate associations between each candidate covariate and implementation of preventive mass vaccination campaign (PMVC) are presented. Each candidate variable was significantly associated with PMVC implementation, with the exception of the log-transformed human population size (Table B).

| **Variable** | **PRR** |
| --- | --- |
| Log population | 0.83 (0.66 - 1.05) |
| Surveillance quality | 1.21 (1.15 - 1.27) |
| Longitude | 0.43 (0.37 - 0.50) |
| Land cover type | 0.60 (0.45 - 0.81) |
| EVI | 1.02 (1.01 - 1.02) |
| Rainfall | 1.23 (1.11 - 1.38) |
| Temperature suitability | 1.48 (1.26 - 1.75) |

**Table B.** Exposure model: Univariate associations between demographic and environmental variables and implementation of preventive mass vaccination campaigns. PRR: prevalence rate ratio calculated from a modified Poisson regression.

***Univariate association between PMVC and yellow fever outbreak in the cohort-style analysis***

Based on a cohort-style analysis, we assessed the univariate associations between the outcome, the occurrence of yellow fever outbreak, and the exposure variable (exposure to PMVC) as well as with candidate confounding factors (Table C). In univariate analysis, exposure to PMVC was not significantly associated with the occurrence of outbreak.

| **Variable** | **uIRR** |
| --- | --- |
| Exposure to PMVC | 0.71 (0.34 – 1.51) |
| Log population | 2.23 (1.54 – 3.22) |
| Surveillance quality | 0.86 (0.69 – 1.07) |
| Longitude | 0.76 (0.65 – 0.90) |
| Land cover type | 0.59 (0.38 – 0.92) |
| EVI | 1.24 (1.03 – 1.50) |
| Rainfall | 1.28 (1.08 – 1.5) |
| Temperature suitability | 0.99 (0.97 – 1.00) |

**Table C.** Univariate associations between PMVC, demographic and environmental variables and yellow fever outbreak. uIRR: univariate incidence rate ratio.

***Multivariate association beween PMVC and yellow fever outbreak in the cohort-style analysis***

Table D presents the complete results (ie including covariates) for the cohort model 1 and cohort model 3 from the main text.

| **Model** | **Variable** | **aIRR** |
| --- | --- | --- |
| Statistical model (cohort model 1) | Exposure to PMVC | 0.37 (0.15 – 0.92) |
|  | Log population | 3.20 (1.87 – 5.47) |
|  | Surveillance quality | 1.09 (0.89 – 1.34) |
|  | Longitude | 0.62 (0.48 – 0.80) |
|  | Land cover type | 0.44 (0.21 – 0.94) |
|  | EVI | 1.63 (1.29 – 2.05) |
| Mechanistic model (cohort model 3) | Exposure to PMVC | 0.65 (0.26 – 1.65) |
|  | Log population | 2.16 (1.32 – 3.51) |
|  | Surveillance quality | 0.99 (0.77 – 1. 26) |
|  | EVI | 1.27 (0.85 – 1.91) |
|  | Rainfall | 1.17 (0.92 – 1.48) |
|  | Temperature suitability | 1.01 (0.98 – 1.04) |

**Table D.** Multivariate association between PMVC, demographic and environmental variables and yellow fever outbreak, according to a statistical and a mechanistic model. aIRR: adjusted incidence rate ratio.

**ADDITIONAL REFERENCES**

1. Garske T, Van Kerkhove MD, Yactayo S, Ronveaux O, Lewis RF, Staples JE, et al. Yellow Fever in Africa: estimating the burden of disease and impact of mass vaccination from outbreak and serological data. PLoS Med. 2014;11: e1001638. doi:10.1371/journal.pmed.1001638

2. Hamlet A, Jean K, Perea W, Yactayo S, Biey J, Kerkhove MV, et al. The seasonal influence of climate and environment on yellow fever transmission across Africa. PLOS Neglected Tropical Diseases. 2018;12: e0006284. doi:10.1371/journal.pntd.0006284

3. United Nations, Department of Economic and Social Affairs, Population Division, Population Estimates and Projections Section. World Population Prospects, the 2015 Revision. 2015 [cited 14 Jul 2016]. Available: https://esa.un.org/unpd/wpp/

4. Dobson J, Bright E, Coleman P, Durfee R, Worley B. LandScan: A global population database for estimating populations at risk. Photogramm Eng Remote Sens. 2000;66: 849–857.

5. Global Administrative Areas | Boundaries without limits. [cited 20 Sep 2016]. Available: http://www.gadm.org/

6. NASA Land Processes Distributed Active Archive Center (LP DAAC). Land Cover Type Yearly L3 Global 1 km SINGrid (12Q1). Sioux Falls, South Dakota: USGS/Earth Resources Observation and Science (EROS) Center,; Available: http://lpdaac.usgs.gov/get_data

7. NASA Land Processes Distributed Active Archive Center (LP DAAC). Vegetation Indices 16-Day L3 Global 1 km (13A2). Sioux Falls, South Dakota: USGS/Earth Resources Observation and Science (EROS) Center,; Available: http://lpdaac.usgs.gov/get_data

8. Xie P, Arkin PA. Analyses of Global Monthly Precipitation Using Gauge Observations, Satellite Estimates, and Numerical Model Predictions. J Climate. 1996;9: 840–858. doi:10.1175/1520-0442(1996)009<0840:AOGMPU>2.0.CO;2
