# Supplementary material for: Assessing the impact of preventive mass vaccination campaigns on yellow fever outbreaks in Africa: A population-level self-controlled case series study
Source: PLoS Med. 2021 Feb 18;18(2):e1003523. doi: 10.1371/journal.pmed.1003523 (PMC7932543; doi:10.1371/journal.pmed.1003523)
Supplement: Attachment — Submitted filename: Response to the reviewers.docx [file pmed.1003523.s009.docx]

Requests from the editors:

*At this stage, we ask that you include a short, non-technical Author Summary of your research to make findings accessible to a wide audience that includes both scientists and non-scientists. The Author Summary should immediately follow the Abstract in your revised manuscript. This text is subject to editorial change and should be distinct from the scientific abstract. Please see our author guidelines for more information: <https://journals.plos.org/plosmedicine/s/revising-your-manuscript#loc-author-summary>
We added the Author Summary in the revised version of our article.

*In the manuscript, please clarify if the analytical approach followed here corresponded to one laid out in a prospective protocol or analysis plan? Please state this (either way) early in the Methods section.

We included a statement about that issue in the Method section:

*“This study relied on datasets that were previously collected and regularly updated for a broader project aiming at estimating the burden of yellow fever and the impact of vaccine activities [4,6]. The analytic plan was defined before the start of the analysis.”*

a) If a prospective analysis plan (from your funding proposal, IRB or other ethics committee submission, study protocol, or other planning document written before analyzing the data) was used in designing the study, please include the relevant prospectively written document with your revised manuscript as a Supporting Information file to be published alongside your study, and cite it in the Methods section. A legend for this file should be included at the end of your manuscript.

b) If no such document exists, please make sure that the Methods section transparently describes when analyses were planned, and when/why any data-driven changes to analyses took place. 

c) In either case, changes in the analysis-- including those made in response to peer review comments-- should be identified as such in the Methods section of the paper, with rationale.

*There are a few places in the text where causal language is used that may not be merited given the possibility for the results to be driven in part by residual confounding and other biases - eg, one such example - Results, "SCCS analysis" section - "Doing so, we estimated that a 10%-increase in vaccine coverage decreased the risk of outbreak by 41% (IRR 0.59; 95% CI 0.46 – 0.76)." - could be better phrased as "10%-increase in vaccine coverage was associated with a 41% decrease in the risk...". There may be other places in the text where similar phrasing needs to be changed.
We corrected such formulation implying causality (see lines…).

-----------------------------------------------------------

Comments from the reviewers:

Reviewer #1: "Assessing the impact of preventive mass vaccination campaigns on yellow fever outbreaks in Africa : a population-level self-controlled case-series study" studies the association between yellow fever outbreaks and preventive mass vaccination campaigns (PMVCs), in selected African provinces from between 2005 and 2018. The primary analytic method used is the self-controlled case series (SCCS), in which each individual province is used as its own control. This allows all time-invariant confounding variables for each province to be automatically accounted for, and is a novel application towards public health intervention, according to the authors. Together with various secondary analyses (summarized in Table 1), it is estimated that PVMCs reduced yellow fever outbreaks by about 34% over the study period.

However, one trade-off of SCCS - other than time-varying confounders - is that the derived conclusions can vary significantly depending on the start and end times of the SCCS analysis (refer for example "Self-Controlled Case Series Methodology", Annual Review of Statistics and Its Application, Whitaker & Ghebremichael-Weldeselassie, 2019). The possibility of such start/end date bias, as well as some other pertinent considerations, might be addressed by the authors to lend further support to their findings:

1. As mentioned, a major concern is that the choice of start and end dates appears able to significantly affect the conclusions. In particular, from Figure 2, half of the pre-PVMC outbreaks occured in 2005 & 2006, with the other half occuring from 2007 to 2014. Although these provinces perform PVMC at different times on or after 2007, the 2007-and-after pre-PVMC incidence of yellow fever is roughly half of the pre-2007 period (13 cases in 116 province-year units, as compared to 13 cases in 66 previously), which may already be suggestive of the aforementioned time-varying confounders.

Moreover, consider if the start date was chosen to be 2007, or the end date as 2015 instead of 2019; it appears that the changes in IRR and T for Equation (1) would lead to possibly significant changes in the calculated prevented fraction, despite the underlying phenomena being the same.

The authors might discuss if there was any particular reason behind the choice of the 2005 start date (e.g. other than to be timed with the beginning of the first Yellow Fever initiative, from Line 330), or if an earlier start date might have been selected to provide better estimates for pre-PVMC incidence rate (from a longer sampling period). Relevant to this, secondary analyses ablating the analysis start and end dates might also be appropriate.

We are thankful to R1 for pointing out this relevant issue. We included a statement justifying our choice of the 2005 start date:

*“In 2005, the African Regional Office of WHO established a yellow fever surveillance database across 21 countries in West and Central Africa based on reports of suspected yellow fever cases [4]. This is likely to have influenced the standards of yellow fever surveillance in these countries. In order to reduce the possible effect of time-changing surveillance quality, the beginning of the study period was set at this date.”*

Moreover, following the R1’s suggestion, we conducted several analyses assess the sensitivity of the SCCS results to the choice of the study period.

*“Furthermore, to assess how the choice of the study period influenced our results, we also conducted a sensitivity analysis considering alternative start and end dates : i) Jan 1st 2007 to Dec 31st 2018 and ii) Jan 1st 2007 to Dec 31st 2014.”*

Results of these sensitivity analysis showed that, when disregarding the 2005-2006 period during which a substantive fraction of pre-PMVCs outbreaks occurred, exposure was associated with a reduced risk of outbreak, and not significantly different as the results observed in the main analysis (IRR= 0.28, 95% CI 0.11-0.70 in the sensitivity analysis versus IRR = 0.14, 95% CI 0.06-0.34 in the main analysis).

Changing the observation period did change our estimates of number of outbreaks prevented as per Equation (1), but once compared to the observed number of outbreak over the same period, the percentage of outbreaks prevented did not change substantially.

The results of this sensitivity analysis are now presented in the new Supplementary Table S3 and referred in the main text:

*“The negative association between exposure to PMVCs and outbreak remained significant across a range of assumptions regarding the imputation of the date (within the same year) of PMVCs implementation and outbreak starting date (when missing), and across various observation periods (Supplementary Table S2 and S3).”*

2. The completeness of outbreak/PMVC data might be further commented on; in Line 134, it is stated that "Outbreak reports that could not be located at the province level were excluded". How common is this occurence? From Line 135 on, it seems that PMVC data was based on two campaigns (the Yellow Fever initiative and EYE strategy). Would almost all PMVCs be reasonably expected to be covered in these sources?

We included a statement describing the occurrence of unsuccessfully located outbreaks:

*“A total of 96 outbreaks out of 97 records (99.0%) were geographically resolved.”*

Regarding the completeness of the data regarding occurrence and localization of PMVCs, it would be indeed highly unlikely that PMVCs occurred over the study period without being tracked by the WHO International Coordinating Group (ICG) on Vaccine Provision. We included a statement in that sense:

*“This virtually ensured completeness of information regarding mass vaccination activities.”*

3. Also, YF mass vaccinations appear to have been common in many African regions from the 1930s through the 1960s (<https://www.who.int/csr/disease/yellowfev/massvaccination/en/>, also Line 331), and possibly thereafter. This appears to be a plausible important confounder.

Indeed, historical vaccination activities may potentially act as a confounder. This confounder is probably time-varying as the contribution of older people (those potentially exposed to these historical campaigns) to the population-level immunity may decrease over time by population renewal.

To assess the strength of such a possible bias, we aimed at quantifying the time-changing proportion of the population that may have been exposed to these historical campaigns (roughly, adults born up to 1960). We relied on demographic data from the World Population Prospect to evaluate the country-specific proportion of adults born up to 1960 (Figure R1). Over the observation period, this proportion decreased by approximatively 5 to 7%. This means that, under the majoring hypothesis that historical vaccination campaigns reached 100% of the population, the population-level immunity in the countries present in the SCCS sample would have decrease by 5 to 7% between the beginning and the end of the study period. We thus believe that the potential confounding bias associated with such a reduced time-changing population-immunity would be limited.

Moreover, if existing, this confounding bias is likely to have led to an under-evaluation of the preventive effect of PMVCs on outbreak risks. Indeed, decreasing population-level immunity would have increased the risk of outbreak during the more-recent period, which corresponds to the exposed period.

These elements thus rather suggest that historical vaccination activities may not translate into a confounding phenomenon that would largely affect our results.

Figure R1: Country-specific time-varying proportion of adults born up to 1960 among the total population in 13 countries

4. The definition of "yellow fever outbreak" does not appear to be defined in the paper, and is only described as being compiled from the WER and DON. The specific criteria (e.g. Percentage of cases increase over some period? Percentage of population affected?) might be explicitly stated.

We clarified the definition and procedure for outbreak investigation in the Method section.

*“As per WHO recommendations, a single, confirmed yellow fever case is sufficient to justify outbreak investigation. Based on the results of the investigation and on the absence of other suspected cases, an alert can be classified as an isolated case, which was not considered for this study [17].”*

5. Also, it seems possible that some outbreaks may be significantly more serious/widespread than others; the provinces themselves appear to possibly be of widely different sizes/populations, such that near-concurrent outbreaks in multiple smaller provinces, might have been judged to be a single outbreak were they a single province. While this appears partially addressed by analysis on spatial autocorrelation, it would be good if the differing scales of outbreaks might be accounted for.

While the final sizes of yellow fever outbreaks is sometimes available in epidemiological records, it is usually acknowledged that this information is often of poor quality, and likely to be largely underestimated. Indeed, the symptoms spectrum associated with yellow fever is wide and rather non-specific. Moreover, the African capacities to laboratory confirm suspected cases were very limited over the largest part of our study period (they are currently increasing as per the EYE strategy launched in 2017). Therefore, we preferred to treat the information regarding outbreaks as qualitative (presence/absence) rather than quantitative. This choice is current when analysing yellow fever outbreaks at large scale (see refs 4 to 6 in our main text).

6. In Line 165, "categories with 20% bandwidth" might be explained further.

We clarified this point:

*“…we considered the estimated population-level vaccine coverage as an alternative time-varying, quantitative exposure (considered as categories with 20% bandwidth: 0-19% / 20-39% / 40-59% / 60-79% / 80-100%).”*

7. More details about the vaccinations might be provided if possible. Was the same vaccine (17D?) used for all provinces, were there any variations (e.g. fractional doses)? Would it be common for some provinces to have had ad-hoc vaccination/vaccination in infants?

We provided more details in the Method section regarding PMVCs:

*“Note that information on the vaccine strain used for each PMVCs are not widely available, however, both vaccine strains recommended by the WHO (17D and 17DD) do not differ in terms of efficacy [13,19]. PMVCs considered here implied full-dose vaccine only as fractional-dose vaccination has been recommended by WHO only in the context of outbreak response [20].”*

8. The correspondence between an estimated prevented fraction of 22%-45%, and an aversion of 28-80 outbreaks, might be described in further detail.

We added a sentence describing further the detail of the calculation (note that we can also explicitly refer to the Equation 3 here if the reviewer or the editor judges it useful):

*“When considering a total of 96 outbreaks occurring over the considered time period, this corresponds to a prevented fraction of 34% (22% to 45%).”*

9. In Table 2, the Unexposed (Ref.) of 32 for SCCS Model 2 might be further explained.

Considering all outbreaks over the study period instead of the first one only (SCCS model 2) allows accounting for multiple outbreaks within single provinces. In this situation, secondary or tertiary outbreaks could occur either before or after PMVCs (ie during the unexposed or exposed period, respectively). This explains why the number of outbreaks for both unexposed and exposed periods are higher as compared to SCCS Model 1. To clarify the difference between SCCS models 1 and 2, we added the mention “1st outbreak only” for the SCCS model 1 in the Table 2.

10. There are some minor grammatical issues (e.g. "started few time", Line 330; "28 to 80 outbreak", Line 351)

We corrected these typos.

In summary, historical experience seems to broadly support the effectiveness of mass vaccination in preventing yellow fever outbreaks; however, the proposed quantification of this effect as presented in this manuscript through SCCS might stand to be further refined, in particular on potential sensitivity towards the choice of start and end dates.

-----------------------------------------------------------

Reviewer #2: ---
Review comments for manuscript ID: "PMEDICINE-D-20-03496", entitled "Assessing the impact of preventive mass vaccination campaigns on yellow fever outbreaks in Africa : a population-level self-controlled case-series study" of journal "PLOS Medicine".

Comments:
-Overall I think the cohort mechanistic model is more trust-worth, because it used all data (exposed and unexposed) and external factors (which is important).
The question of the relative efficiency of the SCCS method relatively to the cohort method has been extensively analysed. See for example Whitaker et al, *Stat Met Med Res* 2009 or Farrington & Whitaker, *Applied Stat* 2006. These studies showed that the loss of efficiency is small, especially when the proportion of the sample exposed is high (26% in our study). Importantly, this moderate loss of efficiency should be weighed against the fact that time-invariant confounders are implicitly controlled for. In the context where the risk of residual confounding from an indication bias is high, this advantage especially counterbalances the loss of efficiency.

We modified the Discussion section in order to emphasis this argument, and provide an additional example (as reference) in which the results of SCCS, unaffected by indication bias, yielded to more valid results that the corresponding cohort analysis (Kramarz et al, *Arch Fam Med*, 2000):

*“Analysing cases only, instead of the corresponding complete cohort, translates into a loss of efficiency, but previous work showed that this loss is small, especially when the fraction of the sample experiencing the exposure is high [24]. Moreover, this loss of efficiency has to be weighted against a better control of time-invariant confounders. Previous examples illustrated that the SCCS design is likely to produce more trustful results than the corresponding cohort analysis, especially when a strong indication bias is likely [25,26].”*

- I think the SCCS model study focusing on the exposed provinces only is of limited value. Because they ignored the data of unexposed provinces. The potential influences of unexposed provinces on exposed provinces should be considered as well. Because the provinces (neighboring) should impact each other.

Regarding the fact that the SCCS method ignores some data, please see our response above.

Please also note that we addressed the issue of a spatial autocorrelation in a sensitivity analysis. This sensitivity analysis led to similar central estimates for the preventive effect of PMVCs. Lastly, it is worth mentioning that such a bias due to a neighbouring-provinces effect would most likely act as a non-differential miss-classification bias, with unexposed provinces partially protected by their exposed neighbours. If existing, such a non-differential bias would have biased our results toward the null hypothesis (no effect of PMVCs), which would mean our results in favour of a strong negative association between PMVCs and outbreak risk may have been underestimated. If such a bias exists, it would not have affected our conclusions.

- How is the global climate changes on the YF outbreaks. I think unexposed provinces may be used as a ref. Another proxy, may be activity of other mosquito borne diseases.

Although the risk of YF is projected to increase with climate change, there is no evidence to date, to the best of our knowledge, that current levels of climate change has affected the risk of YF in the recent past. In terms of global changes possibly affecting YF outbreak, deforestation appears more immediately and strongly associated with an increased risk of YF outbreaks than climate change. As we explain in our main text:

*“However, these various factors are likely to have increased the risk of outbreaks and the probability of outbreak detection in the recent period, which overlaps with the post-PMVCs period in our study sample. This may have led to an underestimate of the association between PMVC and yellow fever outbreaks.”*

- Figure 2, please consider to add the total of outbreaks for each year as bar plot in the top of the swimmer plot.

We updated the Figure 2 according to R2’suggestion. Thanks for this useful suggestion.

-In all of these models, one would assume all countries or outbreaks are independent from each other? How confident is this assumption? Alternatively, I would think a mechanistic model for a large region (involve the hardest-hit countries) would show the effect of the control measure. Before and after control measure, the number of outbreaks changed over time for the whole region—

Actually, as we are analysing the first outbreak occurring in a province, we are releasing the assumption of independent events, as recommended in the methodological paper cited as Ref [11]. The sensitivity analysis conducted as per the SCCS model 2 relies on the assumption of independent events, and it produced results that were quite similar to the main analysis.

The typos in Figure 1 legend should be corrected.
We corrected this typo.

--I suggest the authors to redraw some time series of outbreaks, for each country, for unexposed countries , for exposed countries.

If we understand well, what R2 suggests here seems similar as a bootstrap re-sampling. This is what we do in the sensitivity analysis assessing a possible bias due to spatial autocorrelation. This sensitivity analysis produced results that were in line with the main analysis.

--I think some references were missing in the current study, see, for instance, <https://www.nejm.org/doi/full/10.1056/nejmp1803433>,
<https://journals.plos.org/plosntds/article?rev=2&id=10.1371/journal.pntd.0006158>,
<https://www.sciencedirect.com/science/article/pii/S1386653214003692>.

We are thankful to R2 for these suggestions. We added the 1st and the 3rd ref suggested by R2. Note that the 2nd reference suggested is beyond the scope of this study as it assesses the impact of reactive vaccination campaigns whereas we are focusing on preventive campaigns.
-----------------------------------------------------------

Reviewer #4: This manuscript by Jean and colleagues uses a newly applied simple empirical method to estimate the effectiveness of preventative vaccine campaigns for Yellow Fever in Africa. The manuscript is well written, analysis clear and I do believe that the results do make a new contribution to the evidence base on effectiveness of the Yellow Fever vaccine. I do, however, have some concerns about the over simplicity of the method and its (previously) untested application to ecological data. Alas addressing many of these comments may be challenging given the limited data available. On balance, I do still think that this analysis is beneficial is appropriately caveated.


Major comments:

The authors state that "To our knowledge, this is the first time a SCCS analysis was conducted at the population level." This does raise some methodological robustness issues that would (in an ideal world) be explored in a more detailed statistical methods paper with a more appropriate dataset. Issues such as ecological fallacy, impact of over dispersed or autocorrelated events may have a significant impact on using the SCCS method at a population level, but with only 33 provinces under observation and only 7 post exposure events in this dataset I am sceptical whether such issues could be investigated. I appreciate that a full methodological deep dive with other datasets is probably beyond the scope of this manuscript, but perhaps the authors might want to downplay the emphasis on this being a proof of principle for the SCCS on population level data and instead emphasis the similarities between this and interrupted time series analyses that have a long history of application
to population-level data.

We agree with R4 that we did not explore in-depth methodology issues related to the SCCS application at the level of populations, and that it would be out of the scope of this study. We acknowledge that the issues mentioned by R4 deserve further investigation, and we modified the Discussion in that sense:

*“The application of the SCCS design at the population level certainly deserves further methodological assessments to ensure its robustness to specific issues when transposing it at the population level, especially those related to over-dispersed or auto-correlated events. We hope that the present work will stimulate further studies characterizing the advantage and downplays of SCCS as compared to more classically used population-level designs, for instance as interrupted time-series.”*

Regarding the risk of ecological fallacy, we do not think it may apply here. Indeed, ecological fallacy arises when one draws conclusion at a level that differs from the level at which data was observed (classically: conclusions drawn at the individual level based on observation obtained at the population level). In our study, the level of the conclusions matches the level of the observations (the risk of outbreak at the province level is negatively associated with the implementation of vaccination campaigns at the province level). Furthermore, regarding the assumption of a Poisson process and the risk of over-dispersed data, please see our response below.

Justifying the time-frame of protection. The analysis assumes PMVCs provide perpetual protection whereas we might assume protection to wane over time as new births and migration increase the susceptible proportion of the population. While I can see the advantages of using as much data as is available, I do think it would help if the authors could provide an a priori statement about the length of duration of protection from outbreaks that PMVCs are intended or expected to confer.

The assumption of long-lasting high levels of immunity after PMVCs may indeed be challenged by population renewal. However, most of the countries included in our analysis included yellow fever vaccination as part of routine infant vaccination. We included this precision in the method section:

*“In other terms, we assumed the campaigns to achieve uniform high coverage in all age groups across provinces and that this high coverage was maintained up to the end of the study period. This assumption of persisting high coverage is further justified by the inclusion of yellow fever vaccine in the Enhanced Program of Immunization of most of the countries in the study region [24]. Routine infant vaccination may not rapidly increase population-level immunity by itself, but does contribute to maintain high levels once achieved by the means of PMVCs.”*

Time-varying confounding- two un/under mentioned time-varying confounders that might be worth thinking about. First, naturally acquired immunity- especially as some of these PMVCs appear to be in response to recent outbreaks - ideally if more data were available accounting for seasonality and multi-annual cycles in outbreak occurrence would be helpful. Second, I assume (not a subject expert) that PMVCs also involve a number of preventative activities beyond vaccination e.g. vector control / environmental clear up, behavioural awareness, etc, etc that could also decrease the risk of an outbreak. Plausible time varying confounder in this case could also lead to an overestimation of PMVC effectiveness which might be worth mentioning.

We did focus our analysis on PMVCs for several reasons, including the fact that these campaigns are not conducted in response to recent outbreaks, but are conducted preventively. On the scale of years, some provinces may have been targeted by reactive vaccination campaigns previously to PMVCs, but this may only concern one province among the SCCS sample (see Figure S1 in the Suppl. Mat., especially the province presenting a growth in population-level coverage in 2006). Moreover, previous vaccination activities such as reactive campaigns are accounted for in the SCCS model 4 treating vaccination coverage as a categorical variable.

Regarding vector control, the EYE strategy document mentions that: “*There is currently a limited public health arsenal of safe, efficient and cost-effective insecticides that can be used against adult vectors. This is mainly due to the resistance of major vectors to common insecticides*” (p19, available at: <https://www.who.int/publications/i/item/9789241513661>). Moreover, as the urban vector of Yellow Fever *Aedes aegypti* bites during daytime, devices like bed nets are not particularly relevant. Therefore, and as the EYE strategy recognises, there are limited prevention options beyond vaccination.

Minor comments:

Suitability of representing outbreaks as a Poisson process- are they not over dispersed? (I appreciate difficult o answer empirically with this dataset)

Based on the entire sample of the 479 provinces, we observed a total of 96 outbreaks over the study period. Under the assumption that these outbreaks are distributed according to a Poisson probability distribution, the corresponding Poisson rate is λ = 96/479. The simulated counts below were obtained from 10,000 random realizations of a Poisson process of rate λ = 96/479.

| **Number of outbreaks** | **0** | **1** | **2** | **3** |
| --- | --- | --- | --- | --- |
| **Observed count** | 398 | 69 | 9 | 3 |
| **Simulated counts** : Median and 95% Confidence interval | 392 (375-408) | 78 (63-95) | 8 (3-14) | 0 (0-2) |

**Supplementary Table S1:** Fit of the Poisson probability distribution to outbreak data.

We provided these results in the Supplementary Material (Table S1) and added a statement in the Results section:

*The Poisson probability distribution applied satisfactorily to the observed outbreaks distribution (Supplementary Table S1).*

Mechanistic model covariates not being associated in this analysis is interesting result- any thought as to why + implications for future mechanistic modelling work on this subject?

The mechanistic model we relied on was fitted to data of presence/absence of yellow fever records that only partially overlapped the presence/absence of yellow fever outbreaks. Indeed, additionally to the localisation of yellow fever outbreaks, the data set to which the mechanistic model proposed by Hamlet et al was fitted to also included localisation of individual isolated cases. As such, the mechanistic model may rather capture the drivers of sylvatic yellow fever circulation, which may subsequently trigger urban outbreaks, while we were only considering the later in our analysis.

-----------------------------------------------------------

Any attachments provided with reviews can be seen via the following link:
