# Supplementary material for: Assessing the impact of preventive mass vaccination campaigns on yellow fever outbreaks in Africa: A population-level self-controlled case series study
Source: PLoS Med. 2021 Feb 18;18(2):e1003523. doi: 10.1371/journal.pmed.1003523 (PMC7932543; doi:10.1371/journal.pmed.1003523)
Supplement: Attachment — Submitted filename: Response to Editors requests.docx [file pmed.1003523.s010.docx]

Requests from Editors:

1.      Please address the final reviewer comments below. We did so (see lines 425-433).

2.      Lines 20-34, and 453-458: These can be deleted – these data (author contributions, competing interests, funding etc.) are taken from the online submission form. We did so.

3.      Abstract:
a.      Lines 42-43: Please reword to “However, by how much PMVCs are associated with a decreased risk of outbreak to occur has not yet been quantified.” (Your study design cannot prove causation.) We did so.
b.      Line 47: Please list the main confounding factors accounted for. We did so.
c.      Line 58: Please also include the total number of countries affected. We did so.
d.      Lines 60 and 63: Please replace “reduced” with “associated with a reduction”, or similar . (Your study design cannot prove causation.) We did so.
e.      Please quantify results with p values, as well as 95% CIs. We added a p-value for the risk reduction metric (86%). Note however that our other main metric, the percentage of outbreaks prevented by PMVC, is a metric representing preventable/attributable fraction, which is usually not associated with p-value (see for example: <https://doi.org/10.1371/journal.pmed.1002619>).
f.      Please present data to 1 decimal place, to match the main Results section. The 86% risk reduction is another way to present an incidence rate ratio of 0.14 ( 86 = 100*(1-0.14) ), and 2 decimals is usual to report rate ratios or risk ratios across all the text. For homogeneity purpose, we thus slightly prefer to present data with no decimal rather than present risk reduction with 1 decimal (which would imply to report rate ratio with 3 decimals).

g.      Please perhaps give the total number of infected individuals for the outbreaks, for reference, if known. This figure is generally unknown, or, when number are given, they are considered as widely uncertain. Please see our response #5 to R1 in the previous review round.
h.      Line 62: Please list the main sensitivity analyses conducted. We did so.
i.      Line 66: Please list another limitation, eg. possible unmeasured confounding factors. We did so.
j.      Conclusion: Please start with “In this study, we observed that…”, or similar. We did so.

4.      Author Summary:
a.      Please check the dates quoted, to match the main text. We did so.
b.      Line 86: Please clarify “exposed and unexposed periods”, “same units of analysis”, and “known and unknown confounders”, for a lay reader. We did so.
c.      Line 91: Please clarify “confounding by indication that was not entirely controlled for”, for a lay reader. We did so (note that indication bias is now explained above).
d.      Line 98: “postponed”. This is now corrected.

5.      Methods: If a prospective analysis plan (from your funding proposal, IRB or other ethics committee submission, study protocol, or other planning document written before analyzing the data) was used in designing the study, please include the relevant prospectively written document with your revised manuscript as a Supporting Information file to be published alongside your study, and cite it in the Methods section on line 160. A legend for this file should be included at the end of your manuscript. The researchers agreed on the analysis plan before the start of the study but no official document has been written at that time.

6.      In the Methods, please state that this study did not require ethical approval. We did so.

7.      Note from the Academic Editor: There is a minor note for lines 182-183 -- that's not exactly what WHO says about fractional dosing. Please do a very thorough review of the language and terms used, due to the precision of epidemiologic concepts used. For example, you state that the efficacy of the YF vaccines is known and the same for both 17D and 17DD -- this isn't quite right, it's not efficacy (there's no efficacy trial of YF vaccines). We are thankful to the Academic Editor for recommanding thorough formulation. “*Efficacy*” has been changed for “*Immunogenicity*” and we reformulated the WHO position: “*fractional-dose vaccination has been approved by WHO only as part of an emergency response to an outbreak if there is a shortage of full-dose YF vaccine*”.

8.      Line 353: Please add “95% CI” in the brackets. We did so.

9.      Figure 2B: If possible, please give the full province and country names on the vertical axis. It was not possible as some province names are quite long. However, we provided a correspondence table as Supplementary Table S2.

10.     Please provide access details (DOI or URL) for reference 19. We did so.

11.     Please mark preprint references as “[preprint]”. We did so. Note that the ref 35 preprint may be published in days, we will let you know.

12.     When completing the STROBE checklist, please use section and paragraph numbers, rather than page numbers. We did so.


Comments from Reviewers:

Reviewer #1: We thank the authors for addressing most of the issues raised in the previous review round, in particular the additional sensitivity analyses. It appears that historical data available for certain details (e.g. size of outbreak) is inherently not extremely reliable, with few prospects of additional clarification. However, acknowledging these caveats, the presented findings appear largely justified.

Nevertheless, the authors might consider explicitly including their treatment of Point 3 in the previous review round (on the [non-]confounding effect of past generations being mass-vaccinated) as an additional limitation, in the revised manuscript. We did so (see lines 425-433).


Reviewer #4: The authors have adequately addressed all my comments.
